# The correlation between body mass index and the effectiveness of non-surgical periodontal treatment in Taiwan population

**DOI:** 10.1007/s00784-024-06077-4

**Published:** 2025-12-03

**Authors:** Kai-Fang Hu, Jwu-Lai Yeh, Ying-Chu Lin, Pei-Feng Liu, Yu-Hsiang Chou, Ching-Jiunn Tseng

**Affiliations:** 1 Institute of Clinical Medicine, National Yang Ming Chiao Tung University, Taipei, Taiwan; 2 Division of Periodontics, Department of Dentistry, Kaohsiung Medical University Hospital, Kaohsiung, Taiwan; 3 Center for Big Data Research, Kaohsiung Medical University, Kaohsiung, Taiwan; 4 Graduate Institute of Medicine, College of Medicine, Kaohsiung Medical University, Kaohsiung, Taiwan; 5 Department of Pharmacology, College of Medicine, Kaohsiung Medical University, Kaohsiung, Taiwan; 6 School of Dentistry, College of Dental Medicine, Kaohsiung Medical University, Kaohsiung, Taiwan; 7 Department of Biomedical Science and Environmental Biology, Kaohsiung Medical University, Kaohsiung, Taiwan; 8 Department of Medical Research, Kaohsiung Medical University Hospital, Kaohsiung, Taiwan; 9 Center for Cancer Research, Kaohsiung Medical University, Kaohsiung, Taiwan; 10 Institute of Biomedical Sciences, National Sun Yat-Sen University, Kaohsiung, Taiwan; 11 Department of Medical Education and Research, Kaohsiung Veterans General Hospital, Kaohsiung, Taiwan; 12 Department of Medical Research, China Medical University Hospital, China Medical University, Taichung, Taiwan

**Keywords:** BMI, Asian, Treatment outcomes, Periodontitis, Non-surgical periodontal therapy

## Abstract

**Objective:**

Obesity and periodontitis are recognized as a major public health problem. The aim of this study was to investigate the response of different body mass index(BMI) groups to non-surgical periodontal treatment(NSPT).

**Methods:**

We retrospectively reviewed the medical records, including baseline medical information and BMI, of patients with periodontal disease. We also analyzed periodontal indices, including periodontal pocket depth (PD), clinical attachment level (CAL), bleeding on probing (BOP), before and after NSPT to assess the correlation between BMI and the effectiveness of NSPT.

**Results:**

A total of 147 patients participated in this study, including 88 individuals in the normal BMI group with an average age of 49.02 ± 10.05 years and 59 individuals in the high BMI group with an average age of 48.15 ± 8.78 years. The results showed that the periodontal index of the abnormal BMI group was higher than that of the normal BMI group both before and after NSPT. NSPT had significant effects on patients with periodontitis in both the normal and abnormal BMI groups. The abnormal BMI group exhibited a significantly higher change in the percentage of PD ≧ 4 mm compared to the normal BMI group (*p* = 0.019).

**Conclusion:**

Despite the fact that Taiwan individuals have lower BMI groupings, those with abnormal BMI exhibit less favorable periodontal indices compared to those with normal BMI. However, NSPT has shown promising results in improving periodontal disease among individuals with abnormal BMI. Encouragement should be given to individuals with generalized severe periodontitis and a high BMI to actively consider receiving periodontal treatment.

**Clinical relevance:**

Despite generally lower BMI in Taiwan, individuals with abnormal BMI exhibit less favorable periodontal health. NSPT shows promise in improving their periodontal condition. Tailored interventions may enhance periodontal outcomes.

## Introduction

Obesity is recognized as a major public health problem and it is a complex condition associated with various systemic health issues, including diabetes, cardiovascular diseases, and inflammatory disorders [1]. These conditions can contribute to an increased risk of developing periodontal disease or exacerbate existing periodontal conditions. The causes of obesity are multifactorial and include lifestyle, diet, environment, and genetic factors. Previous data showed that a dysbiotic intestinal microbiota is involved in the development of obesity [2, 3] and characterizes people with obesity [4]. Dysbiosis of the oral microbiota leads to an organizational change in this ecosystem, which can potentiate the effect of specific species such as anaerobic Gram-negative bacteria [5–7]. It is worth noting that the presence of elevated levels of Gram-negative bacteria is closely linked to the onset and progression of a particular oral condition known as periodontitis. Periodontitis is a chronic inflammatory disease of infectious nature, characterized by the gradual and irreversible destruction of the tissues surrounding the teeth, ultimately resulting in tooth loss [8, 9].

Numerous clinical and epidemiological studies have consistently shown a strong link between obesity and periodontal disease. The updated classification of Periodontal and Peri-implant Diseases and Conditions, jointly issued by the European Federation of Periodontology and the American Academy of Periodontology in 2017, specifically emphasizes the significant impact of obesity on periodontal attachment loss through the mechanism of periodontal inflammation [10, 11]. Individuals who are with obesity face an increased likelihood of developing severe periodontitis. Conversely, there is a correlation between higher body mass index (BMI) and the presence of periodontitis in individuals [12–14]. BMI and obesity exhibit a robust correlation with the occurrence, extent, and seriousness of periodontitis.

Non-surgical periodontal therapy(NSPT) continues to be the established and preferred approach for managing periodontitis. NSPT entails the mechanical elimination of bacterial biofilm and deposits through scaling and root planing, establishing a local environment and microbiota that promote periodontal health. NSPT leads to the substitution of inflamed periodontal tissue with well-vascularized and collagen-rich connective tissues [15, 16]. NSPT has been proven to be effective in treating periodontitis, with clinical trials showing that it results in decreased inflammation, reduced pocket depth, and enhanced clinical attachment levels [17–20].

Previous studies [21–23] revealed that NSPT resulted in a significantly better clinical periodontal outcome among subjects without obesity than subjects with obesity and indicated that obesity (OB) and overweight (OW) individuals require more intensive periodontal treatment compared to normal-weight individuals [24]. The relationship between obesity and periodontitis has been proposed as a potential risk factor [22]. The relationship between obesity and periodontitis has been identified as a potential risk factor. The connection between obesity and metabolic conditions, including hyperglycemia, is complex, making it challenging to determine their individual contributions to the effects on periodontitis. Recent meta-analyses consistently demonstrate a statistically significant positive association between obesity and periodontitis [12, 14].

A study found that obesity was identified as the second most significant risk factor for periodontitis, second only to smoking [25]. However, there is currently limited research on the effectiveness of NSPT treatment in relation to obesity, particularly in populations with lower obesity rates compared to Western populations. This study examined the predictive impact of OB/OW on the clinical response to NSPT in Taiwanese individuals diagnosed with generalized severe periodontitis. Additionally, the research aimed to investigate the effectiveness of NSPT among patients with varying BMI levels.

## Methods

### Study sample

The study protocol was approved by the Institutional Review Board of Kaohsiung Medical University Hospital (KMUH, KMUH-IRB-20140268 and KMUHIRB-E[I]−20220057). We collected data through retrospective chart review from patients who underwent NSPT at the Division of Periodontics, Kaohsiung Medical University Hospital, between 2010 and 2019. Research was conducted according to the current principles outlined in the Declaration of Helsinki on research involving human subjects, as well as all national legal and regulatory requirement.

According to the Taiwan National Health Service, BMI was measured by weight (kg) divided by height measured per square meter (m^2^). Those with BMI greater than or equal to 18.5 kg/m^2^ and less than 24 kg/m^2^ were classified as normal group, those with BMI greater than or equal to 24 were classified as abnormal group. BMI was recorded on the date before the start of periodontal treatment. Among them, abnormal BMI is further divided into people with overweight (OW), with BMI values greater than or equal to 24 kg/m^2^ but less than 27 kg/m^2^, and people with obesity (OB), with BMI values greater than or equal to 27 kg/m^2 ^(Taiwan Ministry of Health and Welfare's National Health Service https://health99.hpa.gov.tw/onlineQuiz/bmi). The inclusion criteria encompassed Taiwan individuals aged 20 and above diagnosed with generalized severe periodontitis, characterized by probing pocket depths exceeding 5 mm and marginal alveolar bone loss exceeding 30%, with over 50% of teeth affected [26]. Participants were required to be in good overall health, determined through a comprehensive medical history assessment, and should not be regularly taking medications. Exclusion criteria comprised individuals enrolled in weight loss programs, those who had taken antibiotics within the past three months, pregnant or breastfeeding individuals, and those who had received periodontal therapy within 6 months of the study baseline assessment.

### Data collection

**Fig. 1:**
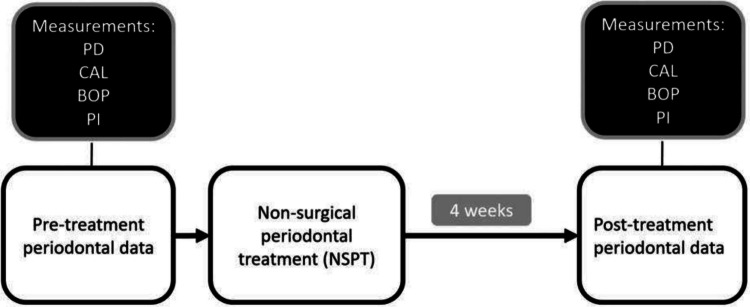
Study flow diagram

The periodontal examination values and BMI were obtained from retrospective medical records. BMI values were calculated based on the individual's height and weight, expressed in kilograms per square meter (kg/m^2^), and measured using a wall-mounted height gauge and mechanical scales. Clinical measurements of the response to periodontal therapy included whole-mouth average probing pocket depth (PD), clinical attachment levels (CAL), the percentage of sites with PD ≧ 4 mm, and the full-mouth bleeding score before and 4 weeks after NSPT [27, 28]. Dental plaque levels and bleeding scores were expressed as a percentage of positive sites for the entire mouth [29]. All clinical variables were assessed at six sites (mesial-buccal, mid‐buccal, distal-buccal, distal-lingual, mid‐lingual, mesial-lingual) per tooth within the oral cavity. All clinical data and NSPT procedures were conducted by trained and certified periodontists. The periodontal data were recorded as follows and Fig. 1:

1. Probing depth (PD, mm): measured from gingival margin to base of the pocket manual periodontal probe (Williams periodontal probe, Hu-Friedy, Chicago, IL, USA).
2. Percentage of sites with periodontal pockets ≧ 4 mm in relation to the total number of sites in the mouth (PD ≧ 4 mm, %)
3. Bleeding on probing (BOP): measured as a dichotomic presence or absence of bleeding 15 s after probing [30]
4. Clinical attachment level (CAL, mm)
5. Percentage of sites with intraoral dental plaque in relation to the total number of sites in the mouth (PI, %) [31]

All patients included in the analysis received non-surgical periodontal therapy, including oral hygiene instructions and full-mouth mechanical periodontal debridement under local anesthesia, performed by the same clinician within a 4-week period using hand and ultrasonic instruments. The patients included in this analysis did not receive any adjunctive procedures, such as locally delivered antimicrobials.

### Sample size calculation

Based on our initial research data, with average PD values of 3.23 mm and 3.51 mm for normal BMI and high BMI, respectively, and setting α at 0.05, power (1-β) at 0.8, employing a two-tailed test, the required sample size for each group was calculated as 52 using the G-power software.

### Statistical analysis

Numerical data of before NSPT (pre-treat) and after NSPT (post-treat) are presented as the mean ± standard deviation (SD), and frequency distributions within each group are indicated with numbers and proportions. Two-sample t test and the chi-square test were used to compare intergroup differences in distribution means and proportions, respectively. Statistical significance was set at *p* < 0.05 and *p* < 0.01. All statistical analyses were conducted using JMP software (SAS Institute Inc., Cary, NC, USA).

## Results

Table 1 is the basic information of this investigated samples, including 88 cases with normal BMI and 59 cases with abnormal BMI. In the normal BMI group, there were 34 males (38.64%), including 6 smokers (6.82%), with an average age of 49.02 ± 10.05 years. In the high BMI group, there were 30 males (50.85%), including 3 smokers (5.09%), with an average age of 48.15 ± 8.78 years. There were no statistically significant differences between the two groups in the above demographic information. The average BMI of the normal BMI group was 21.74 ± 1.36 kg/m^2^, and the average BMI of the abnormal group was 26.92 ± 2.60 kg/m^2^.

**Table 1:** Baseline study sample characteristics (*N* = 147) based on Taiwan ministry of health and welfare national health service category of BMI

	Normal (18.5–23.99 kg/m^2^)	Abnormal (≧ 24 kg/m^2^)	
Variables, Mean (SD)	*n* = 88	*n* = 59	*p*
BMI (kg/m^2^)	21.74 ± 1.36	26.92 ± 2.60	< 0.0001
Age, yrs	49.02 ± 10.05	48.15 ± 8.78	0.590
Gender: Male (%)	34 (38.64)	30(50.85)	0.143
Smoking status: Smoker (%)	6 (6.82)	3 (5.09)	0.667

Table 2 is a comparison of periodontal clinical data between normal BMI and abnormal BMI.

**Table 2:** Comparison of clinical values in normal and abnormal BMI groups

	Normal (18.5–23.99 kg/m^2^)		Abnormal (≧ 24 kg/m^2^)		
	*n* = 88		*n* = 59		
Variables	mean	SD	mean	SD	*p*
pre-treat PD (mm)	3.23	0.59	3.51	0.63	0.006^a^
post-treat PD (mm)	2.84	0.43	3.03	0.45	0.011^a^
ΔPD (mm)	0.39*	0.32	0.48*	0.44	0.164
pre-treat PD ≧ 4 mm (%)	28.17	14.90	36.87	17.41	0.002^a^
post-treat PD ≧ 4 mm (%)	18.23	12.44	22.86	12.10	0.027^a^
ΔPD≧4 mm (%)	9.94*	7.94	14.00*	11.35	0.019^a^
pre-treat BOP (%)	36.72	19.84	38.87	20.80	0.529
post-treat BOP (%)	21.79	13.22	26.16	17.55	0.107
ΔBOP (%)	14.92*	14.13	12.71*	17.74	0.403
pre-treat CAL (mm)	3.80	0.73	4.04	0.76	0.053
post-treat CAL (mm)	3.51	0.68	3.76	0.71	0.035^a^
ΔCAL (mm)	0.28*	0.36	0.28*	0.52	0.964
pre-treat PI (%)	36.25	29.29	39.57	33.56	0.527
post-treat PI (%)	13.30	15.84	20.24	21.67	0.038^a^
ΔPI (%)	22.95*	22.63	19.33*	28.90	0.398

*p < 0.0001 between pre-treat and post-treat

^a^*p* < 0.05 between normal group and abnormal group

### The comparison between normal BMI and abnormal BMI

Regarding the pre-treatment periodontal data, all measured clinical values, including PD (mm), PD ≥ 4 mm (%), BOP (%), CAL (mm), and PI (%), showed higher values in the abnormal BMI group compared to the normal BMI group. The pre-treatment PD (mm) and PD ≥ 4 mm (%) showed a statistically significant difference between the two groups, indicating that the abnormal BMI group had significantly higher PD values and a higher proportion of PD ≥ 4 mm compared to the normal BMI group.

In the pre-treatment periodontal data, all periodontal-related values were higher in the abnormal BMI group compared to the normal BMI group. In the abnormal BMI group, the PD (mm), PD ≥ 4 mm (%), CAL (mm), and PI (%) indices were significantly higher than those in the normal BMI group, with respective p-values of 0.011, 0.027, 0.035, and 0.038. In addition, in terms of the improvement in PD ≥ 4 mm (%), the abnormal BMI group showed a greater reduction in the proportion of PD ≥ 4 mm in the entire mouth after NSPT treatment. The abnormal BMI group experienced a decrease of 14%, while the normal BMI group had a decrease of 9.94%, reaching a statistically significant difference (*p* = 0.019). In terms of the average values of full-mouth PD, the difference in PD before and after NSPT is slightly higher in the BMI abnormal group compared to the BMI normal group, with reductions of 0.48 mm and 0.39 mm, respectively. However, this difference did not reach statistical significance.

The difference in CAL (ΔCAL) was the same for both groups. The improvement ratios of BOP and PI (ΔBOP% and ΔPI %) both improved more in the normal BMI group than in the abnormal BMI group.

### The comparison between OW and OB

**Fig. 2:**
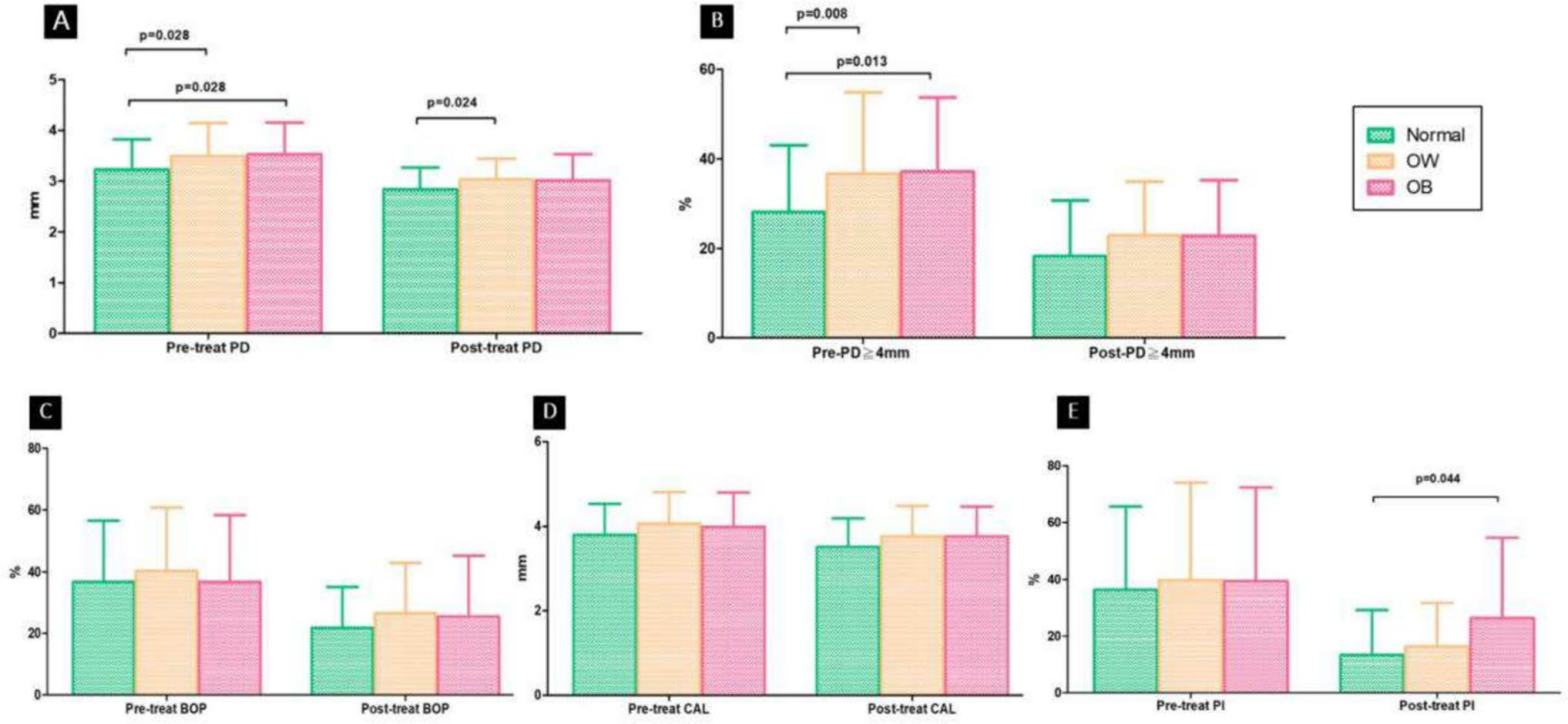
Comparison of periodontal values among the three different BMI groups (normal, OW and OB). OW: people with overweight. OB: people with obesity. **A**: compares the probing depth (PD) values before and after non- surgical periodontal treatment (NSPT); **B**: shows the percentage of sites with a PD greater than 4 mm before and after NSPT; **C**: presents the percentage of sites with or without bleeding on probing (BOP) before and after NSPT; **D**: compares the clinical attachment level (CAL) values before and after NSPT; **E**: illustrates the percentage of sites with or without plaque index (PI) before and after NSPT

**Fig. 3:**
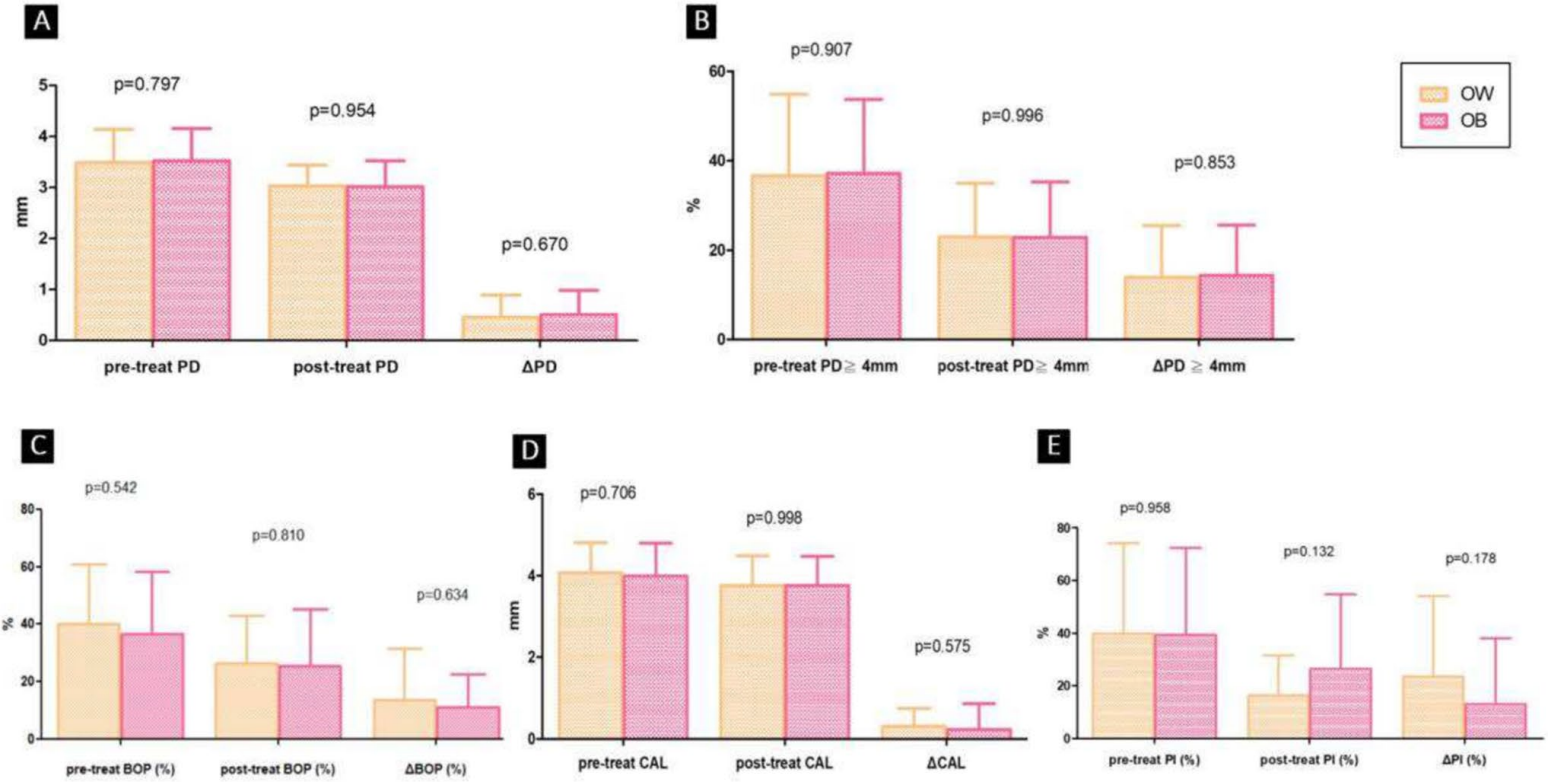
Comparison of periodontal values between the OW and OB groups. OW: people with overweight. OB: people with obesity. **A**: compares the probing depth (PD) values and the improvement before and after non-surgical periodontal treatment (NSPT); **B**: shows the percentage of sites with a PD greater than 4 mm and the improvement before and after NSPT; **C**: presents the percentage of sites with or without bleeding on probing (BOP) and the changes before and after NSPT; **D**: compares the clinical attachment level (CAL) values and the changes before and after NSPT; **E**: illustrates the percentage of sites with or without plaque index (PI) and the changes before and after NSPT

Further classifying abnormal BMI into OW and OB categories for analysis (Table 3), it was observed that the values of PD (mm) and PD≧4 mm(%) before treatment followed a similar trend as BMI values. Higher BMI values were associated with higher average PD values and a higher proportion of PD≧4 mm. Regarding the effectiveness of NSPT, significant differences were observed in all indicators before and after NSPT. This indicates that NSPT has significant effects regardless of BMI level, particularly in the case of PD≧4 mm(%), where significant differences were observed between the OB and OW groups compared to the normal weight group before treatment. However, there were no significant differences among the three groups after NSPT (Fig. 2). Before NSPT, the OB group had significantly higher average PD values compared to the normal weight group (*p* = 0.028). However, after treatment, there were no significant differences between the OB group and the normal BMI group. Furthermore, when comparing the OB and OW groups, which were formed by dividing the abnormal BMI group, it was found that there were no significant differences in the changes of various periodontal indices before and after NSPT between the two groups (Fig. 3).

**Table 3:** Comparison of clinical values in different BMI groups before and after periodontal non-surgical treatment

Group	Normal		OW		OB	
	*n* = 88		*n* = 36		*n* = 23	
Variables	mean	SD	mean	SD	mean	SD
pre-treat PD (mm)	3.23	0.59	3.49	0.65	3.53	0.63
post-treat PD (mm)	2.84	0.43	3.03	0.41	3.02	0.51
ΔPD (mm)	0.39*	0.32	0.46*	0.43	0.51*	0.47
pre-treat PD≧4 mm (%)	28.17	14.90	36.65	18.21	37.20	16.48
post-treat PD≧4 mm (%)	18.23	12.44	22.87	12.10	22.85	12.39
ΔPD≧4 mm (%)	9.94*	7.94	13.87*	11.59	14.35*	11.21
pre-treat BOP (%)	36.72	19.84	40.20	20.55	36.77	21.48
post-treat BOP (%)	21.79	13.22	26.60	16.30	25.46	19.71
ΔBOP (%)	14.92*	14.12	13.6*	18.06	11.31^a^	11.21
pre-treat CAL (mm)	3.80	0.73	4.07	0.74	3.99	0.81
post-treat CAL (mm)	3.51	0.68	3.76	0.73	3.76	0.71
ΔCAL (mm)	0.28*	0.36	0.31*	0.44	0.23^b^	0.63
pre-treat PI (%)	36.25	29.29	39.76	34.31	39.28	33.13
post-treat PI (%)	13.30	15.84	16.35	15.30	26.32	28.31
ΔPI (%)	22.95*	22.64	23.41*	30.71	12.96^c^	25.13

Normal: BMI 18.5 ≦ BMI < 24 kg/m^2^

OW: people with overweight, BMI 24 ≦ BMI < 27 kg/m^2^

OB: people with obesity, BMI ≧ 27 kg/m^2^

^*^p < 0.0001 between pre-treat and post-treat

^a^*p* = 0.003 between pre-treat and post-treat

^b^*p* = 0.046 between pre-treat and post-treat

^c^*p* = 0.011 between pre-treat and post-treat

## Discussion

The results indicated that the periodontal index was higher in the abnormal BMI group compared to the normal BMI group, both before and after NSPT. NSPT had a significant impact on patients with periodontitis in both BMI groups. However, the abnormal BMI group showed a significantly greater reduction in the percentage of sites with probing depth (PD) ≥ 4 mm compared to the normal BMI group.

Previous studies [21–23, 29, 32] have been based on the World Health Organization's BMI criteria. Obesity is defined with a BMI of at least 30.0 kg/m^2^, whereas OW is defined with a BMI of 25–29.9 kg/m^2^. Normal weight is characterized by a BMI ranging between 19 to 24.9 kg/m^2^ [33]. This BMI cut-off points for overweight and obesity are too high for Asian people [10]. In our research, we employed the BMI categories defined by the Taiwan Ministry of Health and Welfare's National Health Service. The standard BMI falls within the range of 18.5 to 23.99 kg/m^2^, while overweight or obesity is categorized as a BMI over 24 kg/m^2^. Given that the prevalence of obesity in our population is not as pronounced as in Western populations, we rely on the BMI classifications established by Taiwan's Ministry of Health and Welfare's National Health Service. This approach is expected to offer a more accurate representation of the obesity landscape among Taiwanese or Asian individuals.

Our study found that both before and after treatment, all periodontal parameters were higher in the abnormal BMI group compared to the normal BMI group, indicating that even after treatment, individuals with abnormal BMI had poorer periodontal health compared to those with normal BMI. Based on previous literature, there have been numerous studies on the PD index, and most of them have indicated that individuals with obesity typically have deeper PD [1, 34–36]. Other periodontal-related indices such as CAL, BOP, and PI showed similar results in a study conducted in 2014 [29], where both pre- and post-treatment periodontal indices were less ideal in individuals with abnormal BMI. However, a study by Benguigui et al. [37] suggested that only PD and PI, among the periodontal indices, were correlated with BMI, while CAL and GI (gingiva index, a periodontal index similar to BOP) were not associated with BMI. The finding that individuals with higher BMI consistently exhibit elevated periodontal indices even after undergoing NSPT indicates that individuals with abnormal BMI continue to have poorer periodontal health compared to those with normal BMI. This suggests that individuals with abnormal BMI may require additional periodontal care. BMI is considered an indicator of overall body obesity, and the high or low values may reflect the general health status. Previous studies have indicated an association between body obesity and the occurrence and severity of periodontal disease [34, 38]. Elevated BMI has been linked to increased inflammation, compromised immune function, and an elevated risk of systemic chronic diseases [39, 40]. Therefore, in the context of a relatively healthy oral environment, further investigation into the implications of BMI levels could provide a deeper understanding, including exploring the interconnection between overall health and oral health. Such research contributes to a more comprehensive comprehension of individual health conditions and the potential impact of BMI on oral health maintenance, thereby offering more effective preventive and therapeutic strategies.

Regarding the effectiveness of NSPT treatment, in terms of the improvement in the percentage of sites with PD ≥ 4 mm, the group with abnormal BMI showed a greater reduction compared to the group with normal BMI after undergoing NSPT. The abnormal BMI group exhibited a decrease of 14%, while the normal BMI group showed a decrease of 9.94%, indicating a statistically significant difference (*p* = 0.019, Table 2). This suggests that although the percentage of sites with PD ≥ 4 mm was significantly higher in the abnormal BMI group compared to the normal BMI group before and after treatment, the abnormal BMI group experienced a greater improvement in reducing the proportion of sites with PD ≥ 4 mm across the entire mouth after undergoing NSPT (14% vs. 9.94%).

Comparison of the improvement in periodontal indices between the normal and abnormal BMI groups showed that only ΔPD≧4 mm(%) reached statistically significant differences. The values for PD and PD≧4 mm(%) indicated that the abnormal BMI group had greater improvement, but there was no statistically significant difference in ΔPD (mm). This suggests that NSPT still has a good effect on improving PD in the abnormal BMI group. Al-Zahrani et al. [22] conducted a PD analysis on female periodontal patients undergoing NSPT and found that obesity did not affect the effectiveness of NSPT. Duzagac et al. [21, 24, 41, 42] also stated that obesity does not affect the effectiveness of NSPT. However, some studies have indicated that a higher BMI may lead to less ideal treatment outcomes with NSPT [1, 32, 43]. While a high BMI, as observed in the patients recruited for this study, may not immediately manifest systemic diseases, its association with elevated levels of inflammation and compromised immune function can impede the desired outcomes of NSPT. Regarding treatment effectiveness, previous research has shown that the correlation between the effectiveness of NSPT and the severity of periodontal disease prior to treatment is much greater than the correlation with obesity levels [1]. A study from 2020 indicated that the plaque index and gingival bleeding index before treatment can reflect the existing inflammatory condition [44]. The plaque index and gingival bleeding index before treatment may serve as reference indicators for treatment effectiveness. In our study, the values of PI (%) and BOP (%) between the normal BMI and abnormal BMI groups were comparable. The normal BMI group had PI of 36.25% and BOP of 36.72%, while the abnormal BMI group had PI of 39.57% and BOP of 38.87%. This may also explain why the treatment effectiveness in the abnormal BMI group is not inferior to that in the normal BMI group.

Furthermore, when comparing the OB and OW subgroups within the abnormal BMI group, it was found that there were no significant differences in the changes of various periodontal indices before and after NSPT (Fig. 3). This suggests that there is no significant difference in the response to NSPT between OB and OW individuals. The difference in NSPT effectiveness is only observed between the normal and abnormal BMI groups. Therefore, an increase in BMI may not significantly affect the response to NSPT. The study by Eldin et al. [45] and our research yielded similar results, showing comparable effectiveness of NSPT when comparing the OB and OW subgroups within the abnormal BMI group.

In recent studies [46, 47], various body indicators such as waist circumference and waist-to-height ratio have been used in an attempt to find more precise and sensitive physiological values for predicting periodontal disease compared to BMI. However, the majority of results have shown that these body indicators do not exhibit a higher correlation with periodontal disease compared to BMI. This study explores the relationship between BMI, the effectiveness of NSPT, and various periodontal indices. However, there are still several limitations to this research. First, although the proportion of smokers did not differ significantly between the two groups, it cannot be ruled out that a few smokers may have influenced the study outcomes. Second, the analysis samples were self-reported or based on medical records without detailed medical examinations. Individuals with abnormal BMI often have undetected underlying metabolic conditions such as hypertension or diabetes, which may also affect the study results. Besides, the lack of stage/grade values for periodontal disease diagnosis in the study groups, the statistically significant differences in pre-treatment ≥ 4 mm PD values between the groups affecting standardization, and the retrospective nature of the study rather than a longitudinal methodology. Lastly, the study did not specifically investigate the chronic inflammatory mechanisms linking obesity and periodontal disease. Future research should focus on further clarification and in-depth investigation in this aspect.

## Conclusion

In Taiwan, using BMI categorizations different from the WHO, individuals with high BMI generally have poorer periodontal indices compared to those with normal BMI. Although both groups show significant improvement after NSPT, those with high BMI respond less favorably to treatment. Therefore, individuals with high BMI should prioritize self-care and professional periodontal care. Tailored medical advice, including weight loss, alongside periodontal treatment, can enhance therapeutic outcomes for this group.

## Data Availability

The datasets generated and/or analysed during the current study are not publicly available due patients’ privacy but are available from the corresponding author on reasonable request.
